# The Urgent Call for Improved Healthcare for Children in Palestine Amidst Conflict

**DOI:** 10.7759/cureus.57450

**Published:** 2024-04-02

**Authors:** Sohilkhan R Pathan, Kruti B Sharma, Shaffiq Y Saiyad

**Affiliations:** 1 Clinical Research Services (CRS), Bhanubhai and Madhuben Patel Cardiac Centre, Shree Krishna Hospital and Medical Research Centre, Bhaikaka University, Anand, IND; 2 Chemistry, Ashok & Rita Patel Institute of Integrated Study & Research in Biotechnology and Allied Sciences (ARIBAS), Vallabh Vidyanagar, IND

**Keywords:** humanitarian aid, conflict, healthcare, children, palestine

## Abstract

The ongoing conflict in Palestine has exacerbated an already dire healthcare situation for children, with hospitals and medical facilities struggling to function amidst targeted attacks and limited resources. This editorial highlights the urgent need for improved healthcare infrastructure and access to medical services for Palestinian children, who face disproportionate suffering and trauma. The blockade and restrictions on Gaza further compound the crisis, hindering access to specialized care and essential medications. The international community must prioritize humanitarian aid and support to address these challenges, safeguarding the health and well-being of Palestinian children amid conflict. Long-term solutions are imperative to build a sustainable healthcare system that ensures the rights of all children to access quality healthcare services, irrespective of geopolitical circumstances.

## Editorial

The ongoing crisis in Gaza has inflicted a profound and harrowing toll on the health of its populace. Exacerbating existing health challenges and spawning new ones, the crisis has taken a grave toll on both physical and mental well-being. With the health system already teetering on the brink prior to the conflict, it now faces imminent collapse, significantly imperiling the population's health. Hospitals overwhelmed, movement constrained, and space scarce, the health situation deteriorates alarmingly. Particularly vulnerable are women, children, and newborns, bearing the brunt as casualties and grappling with restricted access to vital maternal, newborn, and child health services. Infrastructure ravaged, facilities rendered non-functional, and basic amenities disrupted, the humanitarian crisis intensifies. Scarce clean water and sanitation heighten infection risks, with diseases like diarrhea and chickenpox surging, while the looming specter of cholera and epidemics looms large. The conflict's psychological scars deepen, impacting reproductive health and fueling long-term mental health concerns, especially among children. Lives hang in the balance, with evacuation orders imperiling the most vulnerable and medical facilities struggling without essential resources. Urgent action is imperative to stem the tide of this burgeoning humanitarian catastrophe, safeguarding the lives and well-being of Gaza's civilians, particularly its most vulnerable members: pregnant women, children, and newborns [1].

The "Nutrition Vulnerability and Situation Analysis - Gaza" report highlights dire conditions in the Northern Gaza Strip, largely isolated from aid for weeks. Screening reveals that 15.6% of children under 2 are acutely malnourished, with nearly 3% facing severe wasting. Urgent action is needed to prevent further deterioration, as food, water, and health services remain critically scarce: 90% of children under 2 and 95% of pregnant/breastfeeding women experience severe food poverty. 95% of households reduce meals, with 64% having only one meal daily. Over 95% of households prioritize food for small children, limiting adults' intake [2]. More than 13,000 children in Gaza have tragically lost their lives since October 7, with many others grappling with severe malnutrition. The United Nations Children's Fund (UNICEF) reports that some are so debilitated they lack the energy even to cry [3].

Access to healthcare has been severely affected by the conflict. The blockade and the war have led to a shortage of medicines and fuel, affecting the functioning of hospitals and other healthcare facilities [4]. In Gaza, the impact of the conflict on healthcare access is profound. With 350,000 individuals grappling with chronic conditions like cancer and diabetes, alongside 50,000 pregnant women struggling to obtain essential care, the situation is dire (WHO, 2024). Particularly concerning is the disproportionate effect on women and girls, as services and protective measures falter, heightening risks of gender-based violence and tensions (WHO, 2024). Furthermore, the imminent arrival of approximately 160 pregnant women daily in the upcoming month raises alarms, given the limited availability of emergency obstetric care and other critical health services (WHO, 2024). This crisis is compounded by the fact that almost two-thirds of health clinics in Gaza are currently non-operational, amplifying the urgency for international intervention and support to mitigate the healthcare fallout (WHO, 2024) [5].

As the conflict in Palestine persists, one of the most vulnerable demographics continues to suffer silently: the children. Amidst the chaos of war, the state of healthcare for Palestinian children has reached a critical juncture, demanding immediate attention and action from the international community. The ongoing violence in Palestine has left children disproportionately affected, with their access to healthcare severely compromised. Hospitals and medical facilities are often targeted, leaving them struggling to function under immense pressure and limited resources. The scarcity of essential medical supplies, including life-saving medications and equipment, exacerbates an already dire situation. Moreover, the psychological toll of living in a constant state of fear and uncertainty takes a heavy toll on Palestinian children. The trauma they endure leaves lasting scars, affecting their mental and emotional well-being long after the conflict subsides. Yet, mental health support and counseling services are woefully inadequate, leaving many children without the necessary support to cope with their experiences.

Furthermore, the blockade and restrictions imposed on Gaza exacerbate the healthcare crisis, preventing many children from receiving critical medical treatment outside the region. The lack of access to specialized care further endangers the lives of Palestinian children with chronic illnesses or injuries requiring advanced treatment unavailable locally. In light of these pressing challenges, the international community must prioritize the provision of humanitarian aid and support to improve the healthcare infrastructure in Palestine. This includes ensuring unhindered access to medical supplies, facilitating the transfer of patients in need of specialized care, and investing in mental health services for children traumatized by conflict. Additionally, concerted efforts must be made to protect healthcare facilities and personnel, as they serve as lifelines for Palestinian children in need of medical assistance. Targeting hospitals and ambulances is not only a violation of international humanitarian law but also deprives innocent children of their right to access essential healthcare services. Beyond immediate relief efforts, long-term solutions are imperative to address the systemic challenges facing the healthcare system in Palestine. This includes investing in education and training for healthcare professionals, upgrading medical infrastructure, and promoting sustainable development initiatives that prioritize the health and well-being of Palestinian children.

In conclusion, the plight of Palestinian children amidst the ongoing conflict demands urgent attention and action from the international community. Ensuring access to quality healthcare is not only a moral imperative but also a fundamental human right that must be upheld and protected, regardless of geopolitical circumstances. It is time for the world to stand in solidarity with the children of Palestine and work toward a future where they can grow and thrive in peace and security.
